# Public Perceptions of COVID-19 Vaccines: Policy Implications from US Spatiotemporal Sentiment Analytics

**DOI:** 10.3390/healthcare9091110

**Published:** 2021-08-27

**Authors:** G. G. Md. Nawaz Ali, Md. Mokhlesur Rahman, Md. Amjad Hossain, Md. Shahinoor Rahman, Kamal Chandra Paul, Jean-Claude Thill, Jim Samuel

**Affiliations:** 1 Department of Computer Science and Information Systems, Bradley University, Peoria, IL 61625, USA; 2 The William States Lee College of Engineering, University of North Carolina at Charlotte, Charlotte, NC 28223, USA; mrahma12@uncc.edu; 3 Department of Urban and Regional Planning (URP), Khulna University of Engineering & Technology (KUET), Khulna 9203, Bangladesh; 4 Department of Accounting, Information Systems, and Finance, Emporia State University, Emporia, KS 66801, USA; mhossai1@emporia.edu; 5 Department of Earth and Environmental Sciences, New Jersey City University, Jersey City, NJ 07305, USA; mrahman1@njcu.edu; 6 Department of Electrical and Computer Engineering, University of North Carolina at Charlotte, Charlotte, NC 28223, USA; kpaul9@uncc.edu; 7 Department of Geography and Earth Sciences and School of Data Science, University of North Carolina at Charlotte, Charlotte, NC 28223, USA; jfthill@uncc.edu; 8 Department of Business Analytics, University of Charleston, Charleston, WV 25304, USA; jim@aiknowledgecenter.com or; 9 E.J. Bloustein School of Planning & Public Policy, Rutgers University, New Brunswick, NJ 08901, USA

**Keywords:** vaccine, sentiment analysis, Public Sentiment Scenarios framework, COVID-19, coronavirus, Twitter, textual analytics, public policy, spatiotemporal analysis

## Abstract

There is a compelling and pressing need to better understand the temporal dynamics of public sentiment towards COVID-19 vaccines in the US on a national and state-wise level for facilitating appropriate public policy applications. Our analysis of social media data from early February and late March 2021 shows that, despite the overall strength of positive sentiment and despite the increasing numbers of Americans being fully vaccinated, negative sentiment towards COVID-19 vaccines still persists among segments of people who are hesitant towards the vaccine. In this study, we perform sentiment analytics on vaccine tweets, monitor changes in public sentiment over time, contrast vaccination sentiment scores with actual vaccination data from the US CDC and the Household Pulse Survey (HPS), explore the influence of maturity of Twitter user-accounts and generate geographic mapping of tweet sentiments. We observe that fear sentiment remained unchanged in populous states, whereas trust sentiment declined slightly in these same states. Changes in sentiments were more notable among less populous states in the central sections of the US. Furthermore, we leverage the emotion polarity based Public Sentiment Scenarios (PSS) framework, which was developed for COVID-19 sentiment analytics, to systematically posit implications for public policy processes with the aim of improving the positioning, messaging, and administration of vaccines. These insights are expected to contribute to policies that can expedite the vaccination program and move the nation closer to the cherished herd immunity goal.

## 1. Introduction

The novel coronavirus disease 2019 (COVID-19) has already claimed the lives of more than 622,825 Americans, and the numbers continue to rise, albeit at a pace that is considerably lower than at the peak of the pandemic (7 July 2021—[1]). This is an acute health crisis in the history of humankind, with more than 185.5 million confirmed cases and 4 million deaths worldwide as of 7 July 2021 [2]. The effects on public and mental health, social structures, and local and global economies have been tremendously disruptive in almost every country [3,4,5].

Vaccines have the potential to catalyze the development of herd immunity (herd immunity is a form of indirect protection from an infectious disease when a sufficient number of people in a community become immune to an infection [6]) and stop the viral COVID-19 rampage. According to early stage research, to achieve herd immunity in the US, 58–85% adults need to be fully vaccinated with a 95% vaccination efficacy (Two mRNA vaccines: Pfizer-BioNTech and Moderna have shown 95% and 94.1% efficacy, respectively, in clinical trials [7,8]). Given the estimate that vaccinations can only reduce 75% of transmissions, achieving herd immunity will require higher proportions (78–94%) of the population to be vaccinated [9]. So far (as of 11 July 2021), according to the US Centers for Disease Control and Prevention (CDC), 58.7% of US adults (age ≥18) are fully vaccinated.

**Figure 1:**
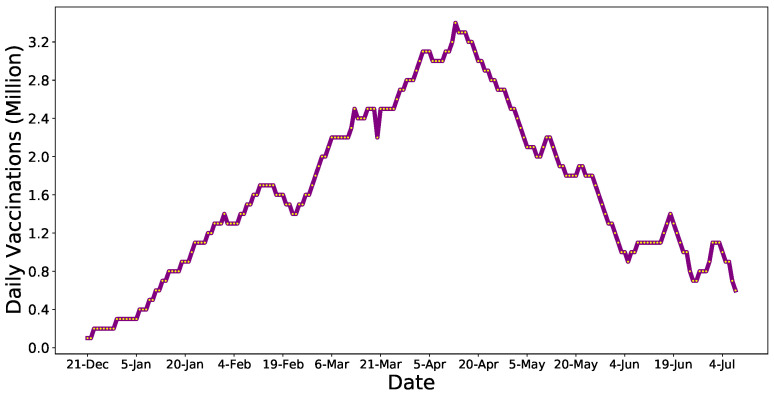
The total number of daily vaccinations (million) in the US (Data source: [12]).

However, it is alarming that the number of doses administered daily has been falling (Figure 1) and that millions of Americans appear to have forfeited or indefinitely postponed the second round of vaccine doses [10]. A recent survey estimated that 40% of adults are hesitant to take a COVID-19 vaccine [11]. This huge number of vaccine-hesitant adults may well prove to be detrimental to the objective of herd immunity induced by vaccination.

However, we noticed a fast rise in vaccine administration in the early period of vaccination (from late December to early April) (Figure 1). Concurrently, a decreasing trend in the daily coronavirus cases and deaths has been observed, particularly in early February 2021 and late March 2021 [1]. The statistics showed a 28.57% and 12.78% reduction in COVID-19 cases and deaths, respectively, on 10 February compared to 1 February. Similarly, an 8.94% reduction in deaths was reported on 1 April over 25 March, despite a 22.42% increase in cases.

Considering the urgency of the situation, this study aims to evaluate public sentiment towards COVID-19 vaccines and to propose policy improvements for organizations, and state and federal governments to facilitate herd immunity and public health enhancements. To accomplish this, we identify public sentiment towards vaccines with Twitter data in the US. Our research studies the progression of public sentiment towards vaccination from early February 2021 to late March 2021 in the US as a whole as well as on a state by state basis.

Insights from Twitter data were verified and validated by the actual vaccination data collected from the CDC [12] and the Nationwide Household Pulse Survey (HPS) conducted by the US Census Bureau from 14 April to 26 April 2021 [13]. The principle values and findings of this research have significant implications for public policies of state and federal governments to expedite the vaccination program and achieve the cherished herd immunity goal.

Public policy has played a dominant as well as contentious role in some countries in addressing the COVID-19 crisis. The success experienced by countries across the world has depended on the effectiveness of their COVID-19 public policies pertaining to healthcare, communication, education, motivation and non-pharmaceutical interventions (NPIs), such as social distancing. Given that the COVID-19 vaccine was not available in the early stages of the outbreak, public policies initially focused on various NPIs (e.g., lockdown, restrictions on mass gathering, bans on travel, border closing, testing, and contact tracing), and economic stimuli (e.g., donations, loans, and debt relief) were implemented to contain the pandemic and mitigate the associated risks [14,15,16,17,18].

Simultaneously, scientists and researchers worked on developing COVID-19 vaccines, which are critical to control virus diffusion, protect human lives, and ensure social and economic recovery [14,16]. The success of the COVID-19 vaccine production, distribution, and actual administration ultimately depends on the public acceptance of the vaccine amid viral misinformation, hesitancy, and fears of side effects and allergies [19,20,21,22].

To reach herd immunity through vaccination, there are two considerations: (1) ensuring the availability of vaccines for all and (2) administering the vaccine. Having a sufficient vaccine supply for the population and having lowered the financial barrier to obtain the vaccine (vaccination is free for all in the US), the greatest challenge then comes in administering the vaccine to enough people due to other common impediments, such as vaccine hesitancy. Usually, developing a vaccine is time consuming and resource intensive. Hence, the prompt availability of vaccines has raised concerns among citizens about their safety and effectiveness [14].

Moreover, vaccine hesitancy is influenced by the level of confidence (i.e., lack of trust in vaccines, in providers, in regulatory authorities, and in the government at large), complacency (i.e., no perceived need for a vaccine and no perceived value), and convenience (e.g., difficult access to vaccines, difficulty to schedule shots, and perceived cost) [14,23,24,25,26]. Researchers found that COVID-19 vaccine hesitancy is very high in many countries due to misinformation about side effects, accelerated research, development and production, lack of trust, and doubt in efficiency [23,27]. Thus, identifying the reasons for vaccine hesitancy and information situation for this position is an important research direction for increasing the COVID-19 vaccine uptake.

**Figure 2:**
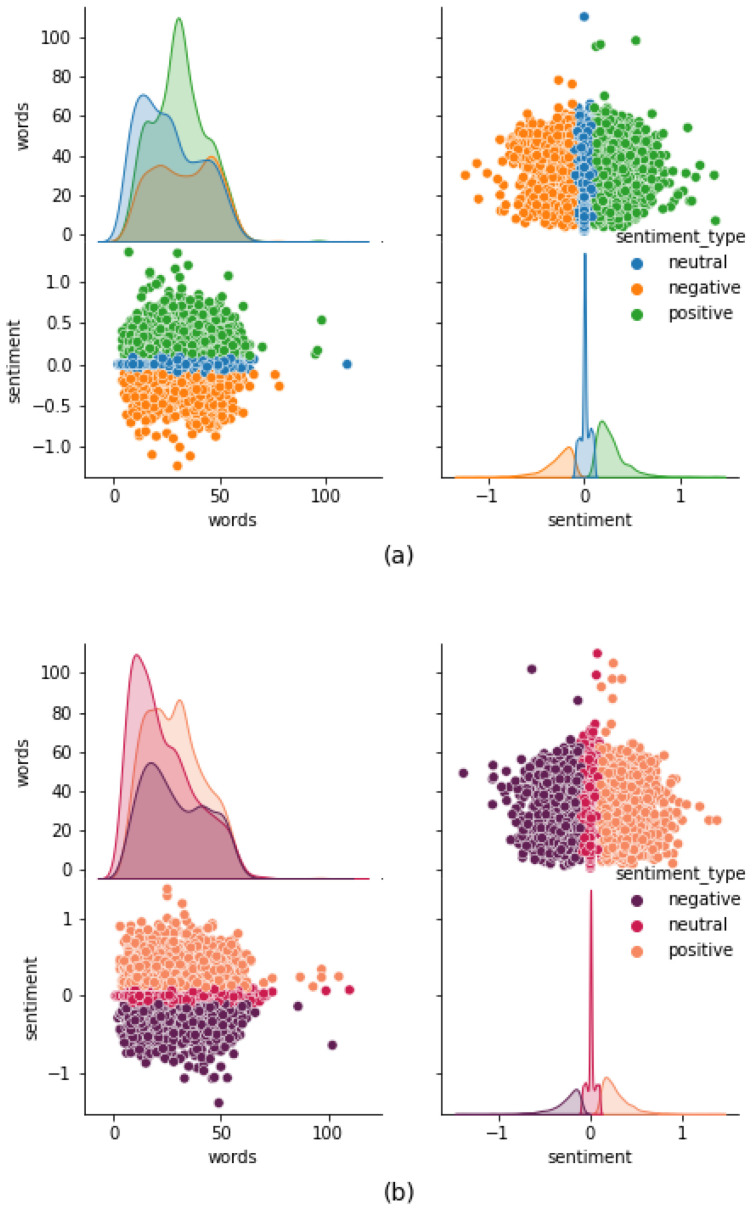
Characteristics of vaccine tweets. (**a**) Tweets in early February 2021. (**b**) Tweets in late March 2021.

Public sentiment analysis based on social media data has become the preferred approach to identify and address many trending issues of our society [4,5,28,29,30]. In this research, we analyzed public sentiment vis à vis COVID-19 vaccination using Twitter data (tweets that were posted from across the US). We identified the progression of public sentiment regarding vaccination by analyzing tweets posted in early February 2021 and again in late March 2021. We classified the sampled tweets into three sentiment groups (see Section 3.2 for the description of the methodology): positive sentiment, negative sentiment, and neutral sentiment. Some of the characteristics of these tweets are displayed in Figure 2.

Overall, we found that the positive sentiment dominated over the negative sentiment on vaccination in the US in both time frames. However, we also detected that, over the time span of the analysis, positive sentiment declined (by around 9.5%), and neutral sentiment increased (by almost 9%). Interestingly, this implies that just allowing more time for Americans to bring themselves to take the vaccine is not going to help the US reach the herd immunity goal. Another important finding from this study is that sentiment varied greatly from one US state to another.

We also found that the state-wise sentiment score exhibited a proportional relationship to people’s proclivity towards vaccination. Hence, we argue that public sentiment is a critical dimension of the design of policies effective at delivering results in overcoming a viral outbreak. On this basis, following a Public Sentiment Scenarios (PSS) framework, we recommend several public policies to state and federal governments and we suggest proactive and incentivizing methods and to take actions that are state specific to encourage American adults to get vaccinated so that the country as a whole can reach the herd immunity goal sooner.

The rest of the paper is organized as follows. Section 2 discusses the related work on vaccination sentiment, social media analytics, and COVID-19 vaccine hesitancy and acceptance. Section 3 discusses the methods of data collection from the Twitter social media platform, the data preprocessing, the computation of sentiments from Twitter posts, and finally the spatiotemporal analysis of the sentiments in the population. We articulate the fundamentals of the PSS framework and its merit in Section 4.1 for the purpose of structuring hypotheses for statistical analysis and of evaluating the public policy implications of the statistical sentiment analysis. The detailed results on state-wise spatiotemporal sentiments towards vaccination are presented, analyzed, and discussed in the other parts of Section 4, along with verification and validation with the actual vaccination information as well as the limitations of the study. Section 5 discusses policy implications. Section 6 concludes the study and outlines some future research directions.

## 2. Related Work

A number of studies have already investigated the public perceptions of COVID-19 vaccines around the world. These studies have explored the status of vaccine acceptance and hesitancy of people as well as their associated causes. Some of these studies collected data from social media (e.g., Twitter, Facebook, etc.) and some performed questionnaire surveys to collect data to conduct the studies. Table 1 provides a summary of estimates of the prevalence of polar opposite views of hesitancy and acceptance of the vaccines. The rest of this section discusses the results of previous studies in more detail to better understand the complex diversities of current opinions and positions on vaccination in different contexts of the world and, particularly, the common contexts for the opposing views of hesitancy and acceptance.

### 2.1. COVID-19 Vaccine on Social Media

Sentiment analysis using social media data is increasingly establishing itself as a useful and powerful tool to understand the individual perceptions of important ongoing matters of broad public concern, such as a pandemic, a disaster, climate change, vaccination, and so on. Thus, several previous studies have used Twitter posts to understand public perceptions and associated factors that influence people’s opinions on the COVID-19 pandemic. For example, Samuel et al. [4] analyzed Twitter data to understand the state and evolution of the sentiment of fear that gripped people’s state of mind while COVID-19 hit the US in February 2020. Similarly, just after the first wave of COVID-19 in the US, Samuel et al. [5] and Rahman et al. [18] aimed to gauge public sentiment towards reopening the US economy and the factors that control sentiments towards such moves, respectively.

Twitter data also feature in previous studies that seek to understand people’s perception of vaccines and vaccination as a means to build immunity against a virus. For example, Hilary et al. [30] analyzed over a million vaccine-related tweets posted over the period of 2011 to 2019 to track the public opinion on vaccines. They classified tweets into positive, negative, and neutral attitudes towards vaccination across geographic areas and historical periods.

Salathé and Khandelwal [38] measured the evolution and distribution of sentiments towards the novel influenza A(H1N1) vaccine. Raghupathi et al. [39] used a natural language toolkit (NLTK) on Twitter data to explore patterns and public opinions towards measles vaccination. Thus, Twitter data have been extensively used to gain broad and nuanced knowledge of public perceptions on a number of ongoing issues related to vaccines and vaccination around the world.

For example, Hussain et al. [35] conducted a study to analyze public sentiments towards COVID-19 vaccines in the US and the UK by extracting information from more than 300,000 posts from Facebook and Twitter collected from 1 March to 22 November 2020. Thelwall et al. [28] used tweets to understand what types of perceptions and attitudes are shared on Twitter so that appropriate actions can be taken to stop the spread of misinformation/disinformation. Clarivate’s social intelligence experts [40] analyzed tweets to monitor the evolving perspectives of Americans on COVID-19 vaccines. Lyu et al. [29] explored public opinion on COVID-19 vaccines in the US after collecting data from 20,000 Twitter users. Thus, social media has been extensively used to understand the perceptions and attitudes of people towards the acceptance of a vaccine around the world.

### 2.2. COVID-19 Vaccine Hesitancy

The main hindrance to reaching herd immunity through vaccination is vaccine hesitancy. Given the criticality of this issue, it is necessary to study the major factors that contribute to vaccine hesitancy in people. As indicated in Table 1, some researchers have investigated vaccine hesitancy among Americans. These studies found that 10% to 40% of people in the US are COVID-19 vaccine hesitant. Additionally, Khubchandani et al. [41] mentioned that about 22% of people who have lower levels of education and household income and perceived threats of getting infected are more hesitant to receive COVID-19 vaccines. Similarly, another study [42] reported that about 23% of medical students in the US are hesitant to take the COVID-19 vaccine immediately after approval from the US Food and Drug Administration (FDA). Thus, there is substantial evidence of vaccine hesitancy among people in the US [43,44].

Jeremy et al. [45] conducted four on-line surveys of 5018 French people (18+) on the reasons of vaccine hesitancy in April 2020. The study results indicated that almost 25% of people refused to take the vaccine. Of those who refused the vaccination, about 64.4% mentioned that the vaccines were developed in a rush and could be too unsafe for them, 8% had no trust in vaccines at all, while 27.6% of people did not mention any specific reasons for vaccine rejection. The study also mentioned that people who were ideologically close to governing political parties had a more positive attitude toward vaccines than those who were not.

By analyzing data from an anonymous web-based survey of 3259 individuals in France, conducted from 26 March to 20 April 2020, Detoc et al. [46] reported vaccine hesitancy as the most significant obstacle in COVID-19 vaccination. Results suggested that 77.6% of individuals were certain or probable to undergo vaccination and 47.6% people had a (certain or probable) inclination towards a clinical trial.

A number of studies conducted cross-sectional surveys on vaccine hesitancy in Hong Kong and China. Wang et al. [47] conducted a study on 806 nurses in Hong Kong, China and found that a low level of COVID-19 vaccination intent and a high level of vaccine hesitancy. Wang et al. [48] studied 2047 working individuals in Hong Kong, China about the change of intentions towards COVID-19 vaccines. They found an increase in vaccine hesitancy during the third wave compared to the first wave among survey participants.

Researchers in [49] conducted a study to identify the patterns of vaccine hesitancy among different socioeconomic groups and residency status in Shanghai, China. The survey was conducted on 1021 parents of children aged under 18 through a questionnaire survey. The results showed that about 73.8%, 63.9%, and 52.4% of respondents expressed strong concerns regarding adverse side effects, safety, and effectiveness of vaccines, respectively. Additionally, they noticed that rural individuals who did not have permitted residency (Hukou) in Shanghai showed more concerns about the side effects, safety, and effectiveness of vaccines compared to local residents of Shanghai.

Janessa et al. [36] sought to uncover the reasons for COVID-19 vaccine hesitancy in Canada. Using the Theoretical Domains Framework, they analyzed the contents of 3915 tweets based on knowledge, beliefs on consequences, environmental context and resources, social influence, and emotion. About 48.3% of these tweets expressed concern about safety due to the rapid development of a vaccine, 32.4% were skeptical about the political motivation towards COVID-19 vaccine development, 26.3% of tweets indicated the lack of accurate knowledge of users due to misinformation about COVID-19, vaccination, and herd immunity. In addition, 8.4% of tweets expressed a certain mistrust of the vaccines because government, civic and business leaders were not seen to wholeheartedly embrace vaccination by being good role models and taking the vaccine early on.

### 2.3. COVID-19 Vaccine Acceptance

Studies have investigated the vaccine acceptance level and associated causes in the US [33,50] and abroad [3,23,51]. For example, Kreps et al. [33] conducted a study to evaluate the factors that are associated with COVID-19 vaccination in the US. Creating two contrasted hypothetical vaccine scenarios based on vaccine efficiency, protection duration, major and minor side effects, approval from the FDA, the origin of vaccines, and endorsement, the survey study conducted on 9 July 2020 explored the willingness of 1971 individuals in the sample to receive the vaccine. The study observed that an increase in vaccine efficiency and protection duration, reduction in major side effects, approval from Centers for Disease Control and Prevention and World Health Organization increase willingness to receive a vaccine, while a vaccine originating from outside of the US, particularly from China, reduced the willingness to receive a vaccine.

Conducting an on-line survey, researchers in [31] collected data from 804 adult individuals in the US. The study observed that men, people who are white and non-Hispanic, college graduates, Democrats, married or partnered, people with pre-existing conditions, and persons who had taken the influenza vaccination in 2019–2020 were more likely to be vaccinated against the COVID-19 virus. CNN/MSNBC viewers had higher intentions of getting vaccinated (76.4%) than Fox News viewers (57.3%).

Similarly, an on-line survey study of 2006 people (18+) [34] in the US identified several factors as key indicators of vaccination acceptance, including a recommendation by a healthcare provider, moderate or liberal political leaning, perceived future likelihood of COVID-19 infection, and the awareness of effectiveness of vaccines.

Bell et al. [3] conducted an on-line cross-sectional survey and semi-structured interviews of 1252 parents or guardians aged over 15 who live in England with a child aged 18 months or less. During semi-structured interviews, several participants mentioned that the availability of the vaccine is the only way to end the lockdown regime and return to normal life. The study also mentioned that respondents from Asian, African, Chinese, low income households, and those concerned about the safety and effectiveness of the vaccine and the hasty trial and development of this relatively new vaccine were less likely to accept the vaccines.

Goldman et al. [50] conducted a study in six countries (i.e., the US, Canada, Israel, Japan, Spain, and Switzerland) to understand the willingness of parents with children aged under 18 to accept a COVID-19 vaccine. Data were collected by administering a cross-sectional survey to 2557 respondents from 26 March to 30 June 2020. Survey results indicate that about 43% of respondents were supportive of expedited testing and approval of the COVID-19 vaccine to make it available for use. This study suggested that public health officials and vaccine providers should strive to better understand the views and attitudes of people as well as their personal circumstances to customize their approach to increase acceptance and administration of the vaccine.

Researchers in [19] evaluated the effects of perceived risk of the COVID-19 pandemic (i.e., the probability of infection, severity, and associated worry) and perceived vaccine safety on the acceptance of COVID-19 vaccine. Conducting an online survey, they collected information form 856 participants in Finland. Investigating the factors of vaccine willingness, this study found that perceived vaccine safety was the core factor, which explained 52% of the variation in intention to accept vaccine. In contrast, this study reported no significant association of perceived risk of COVID-19 with willingness to accept vaccine. Thus, to increase the vaccine uptake, it is necessary to increase the perceived safety of the vaccine.

Leng et al. [51] conducted a study in China to determine people’s preference for COVID-19 vaccine acceptance. A total of 1888 responses were collected across six Chinese provinces and found that trust in the vaccine and in the vaccination process is considered one of the most important factors to increase vaccine administration.

Troiano and Nardi [23] reviewed a number of studies on COVID-19 vaccine acceptance across a number of countries and mentioned the most prominent reasons to reject the COVID-19 vaccine included safety concerns, the rush to produce the vaccine, considered uselessness of the vaccine due to harmless effects of COVID-19, lack of trust, doubts about the efficiency, belief in already immunization for influenza-like illness, and unavailability of the vaccine.

Eibensteiner et al. [37] assessed the perceptions of people on the safety and acceptance of the COVID-19 vaccine worldwide. Creating Twitter polls and pinning them to the Digital Health and Patient Safety Platform timeline for 1 week in mid-February 2021, they found that 45.9% of tweeter users agreed on the adequate safety of the COVID-19 vaccine (i.e., all vaccines are safe). In contrast, about 41.7% were uncertain about vaccine safety. Only 5.2% mentioned that the vaccines are generally unsafe for them.

In summary, from the extensive research based on evidence collected from social media and cross-sectional surveys (Table 1), there is strong evidence that 10 to 40% of people are COVID-19 vaccine hesitant and a couple of critical factors are involved with this hesitancy. Some of the identified factors include lack of trust in vaccine development, rush in vaccine development, politics, health side effects of vaccination, and lack of knowledge on vaccines. It is also observed that female, younger generation, Black/Hispanic Americans, low educational attainment, unemployment, not having been vaccinated for influenza-like illness, anti-vaccine attitudes, and religiousness are negatively associated with a willingness to accept vaccination.

Consistently with studies on hesitancy, we also found that between 35% to 85% of people are interested in taking COVID-19 vaccines. Studies mentioned that higher efficiency of vaccines and protection duration, reduction in side effects, increased trust and safety, and approval from concerned authorities motivate people to take COVID-19 vaccines. Moreover, male, white and non-Hispanic individuals, people with high educational attainment, married people, people with pre-existing conditions, concerns about the COVID-19 infection and disease, and those employed in the health care industry were positively associated with vaccine acceptance.

## 3. Methods

Thus far, we have used a broad range of secondary data sources on COVID-19 and on vaccines along with analysis of the extant literature to articulate a descriptive narrative of the complex scenarios surrounding vaccination and herd immunity challenges. Given the complexity of the public response to vaccination and the fluid state of actual vaccination rates across population subgroups, our analyses, results, and discussions are multifaceted and include the following steps, primarily using Twitter data collected for this study, as well as secondary data for mapping and contrasting discussion purposes:

- Qualitative synthesis of vaccine tweets to illustrate the common public sentiment scenarios;
- Statistical analysis to validate the significance of the change in sentiment from February to March of 2021 as an extension of the original PSS framework; and
- Contrast of findings from the analysis of the Twitter data collected for this study with secondary data, namely CDC and HPS data. CDC data are used for geographical state-wise mapping of vaccination rates while HPS data are used to examine the alignment of sentiment towards vaccines.

### 3.1. Data Acquisition and Preparation

We used the rtweet package in R software to download tweets in early February (3–10 February) and late March (25 March–1 April) 2021 via Twitter’s Research Access API, using the keyword filter “*vaccine*”. After cleaning and preprocessing the downloaded data consisting of over a million tweets, and excluding spam by url posts, we further obtained subsets of 5131 and 9036 tweets for each of the two time periods, respectively, by filtering on “United States” as country. In addition to cleaning the data to remove special characters, we also ran a custom algorithm to replace most of the identifiable abusive words in tweets with a unique character string “*7abuvs12304*” with a low likelihood of natural occurrence in tweets.

### 3.2. Sentiment Analysis Methods

We applied the SentimentR package with the Jockers dictionary to compute the sentiment score of each tweet [52]. The tweets were then classified into three sentiment classes on the basis of sentiment scores: positive sentiment (score > 0.10), negative sentiment (score < −0.10) and neutral sentiment (−0.10 ≤ score ≤ 0.10). Sentiment analysis packages in R provide multiple methods for assigning sentiment scores, the simplest of which is a categorical classification into positive (+1), neutral (0), and negative (−1) sentiments [53,54,55].

A slight improvement of posited accuracy is observed with the RSentiment package, which classifies text into six categories (very negative, negative, neutral, positive, very positive, and sarcasm). However, these tend to approximate observed sentiment, and we chose to apply a relatively more accurate method using the SentimentR package, which provides continuous scores from around +1 for positive sentiment to around −1 for negative sentiment. In this method, extremely few tweets tend to be assigned a perfectly neutral score of 0, despite additional tweets bearing neutral sentiment.

In such scenarios, researchers are required to estimate data-specific boundaries to estimate how the “weighting for valence shifters” accommodates neutral sentiment [56]. Our analysis of a sample of the vaccine tweets data indicated that tweets with scores between +0.1 and −0.1 tended to bear neutral sentiment. Hence, we assigned +0.1 to −0.1 of sentiment scores as our data-specific range for determining neutral sentiment.

**Figure 3:**
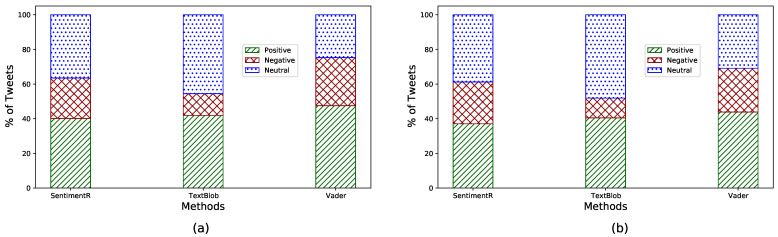
Sentiment classification using three different methods. (**a**) Tweets in early February 2021. (**b**) Tweets in late March 2021.

To further validate the sentiment score calculations, we also used two other lexicon-based Python libraries, namely Textblob and VADER (valence aware dictionary for sentiment reasoning). Textbl provides a simple API for text mining, text analysis, and text processing. It reuses the corpora of the Natural Language Toolkit (NLTK) for text analysis. VADER is another lexicon and rule-based Python library, specifically designed for the analysis of the sentiment of social media text [57]. Oyebode and Orji in [58] showed that VADER performed significantly better in terms of sentiment scoring accuracy compared with Textblob when analyzing social media text. Figure 3a,b depict the comparison of the three methods to identify sentiment classes such as positive, negative, and neutral on the two datasets.

All three methods had a similar trend in classifying tweets. Each method identified a higher percentage of positive tweets over negative tweets with a significant percentage of neutral tweets. Considering the superior accuracy of VADER over Textblob and the similarity of results with sentimentR to identify positive and negative classes, we used the sentiment score obtained with sentimentR in the rest of our analysis.

### 3.3. Word and Phrase Associations

The text corpus created from the filtered and cleaned tweets was used for word and phrase analysis to gain insights into the dominant themes related to COVID-19 vaccination. We used word frequency and N-grams analysis to study the text corpus. Word frequency analysis provides an array of high frequency words, sorted by decreasing frequency. N-grams identify frequently used word pairs and word sequences, which uncovered interesting patterns of association between concepts and themes.

### 3.4. Geo-Tagged Analytics

The US state name of each tweet was determined based on the tagged geo-locations of the tweets within the contiguous United States. Of 5131 tweets in the early February 2021 sample, a total of 5095 had a valid state. Among 9036 tweets in the late March sample, we had a total of 8961 geo-located items. With the Syuzhet package in R, we scored each tweet for eight different sentiments, including anticipation, joy, trust, surprise, fear, sadness, anger, and trust. The aggregated sum of each sentiment score was calculated for each state, and then normalized by the number of tweets of each state. The percentages of positive, neutral, and negative sentiments were calculated at the state level as well. The state-wise sentiment is discussed in Section 4.5.

## 4. Results and Discussion

In this section, using methods posited under the PSS framework [5], we provide sufficient statistical validation of our computed sentiment values. This is followed by descriptive sentiment analytics. We repeat and extend the statistical process used in the original PSS framework, which analyzed sentiment scores and verified data properties with an ordered barplot and Q-Q plots, respectively, and compared the means using proportion and exact binomial tests. Then, we proceed with a descriptive US state-wise sentiment analysis, which is compared to the spatial series of state-wise actual vaccination data in the discussion subsection.

### 4.1. Insights from PSS

The PSS framework was initially developed to analyze COVID-19 public sentiment towards reopening the economy for the purposes of catalyzing and augmenting public policy formulation [5]. Samuel et al. highlighted the tremendous policy value of latent public sentiment and the need to capture it effectively for the purposes of influencing and contributing to the formulation of public policies at all relevant levels, including federal, state, and local governance. They carefully articulated the power of public sentiment, public perceptions, and public opinions and posited that the collective wisdom of mass, public, spontaneous, and continuous-stream social media posts are reflective of such public sentiments, perceptions, and opinions.

They also referenced a broad literature that demonstrates the use of social media analytics to gauge public opinion, and the power of public sentiment towards diverse issues, ranging from the stock market, to judicial processes, political agendas, technological innovation, crisis management policies, and the study of human behavior [59,60,61,62,63].

Social media information is powerful and can pose contagion challenges owing to the virality of posts, as the information content and the format of feeds and posts have been shown to influence human sentiment and actions [64,65]. In the original PSS framework, the scenarios were presented as alternatives based on aggregate public sentiment conditions. In this study, we adapt the PSS theoretical framework and apply the concept to scenarios that may simultaneously emerge and coexist in different sub-populations. We use the PSS framework to structure our hypotheses for statistical analysis, and then to evaluate the public policy implications of the sentiment statistical analysis performed on vaccine tweets.

#### Scenario Analysis: 3 Significant Scenarios

The US is currently (May 2021) in an enviable position globally where, on any given day, it has more vaccines in stock than people willing to receive it. News reports have indicated increased public hesitancy towards the vaccine, partly due to the controversial federal government’s decision to implement a 10-day pause on the use of the Johnson & Johnson vaccine, to some attrition related to people who have already taken the first dose but do not appear to be returning for the second vaccine dose in a timely manner, and to a variety of other considerations.

We use the PSS framework to analyze the dominant sentiment in February and March 2021, and the two significant public sentiment changes—a change in positive sentiment from early February to late March, and change in negative sentiment from early February to late March. We do not dwell upon neutral sentiment in our analysis. The two changes do not automatically correlate negatively as may be intuitively perceived, because it is possible that both positive and negative sentiment counts or scores increase or decrease together at the expense of neutral sentiment. Hence, three scenarios are studied.

(a) **Scenario 1: Positive public sentiment is dominant in February and in March 2021.** Given this scenario, selected on the basis of descriptive analytics, we observe strong positive sentiment towards the vaccine. Illustrative tweets include celebratory posts, such as: *“@PurelyNumbers Yay for vaccine!... ”; “@sprizee Happy birthday and congrats on the vaccine!”; “2nd dose ready. #vaccine #Happy”; “got a pfizer vaccine lined up for 4:20p on the afternoon before my birthday, nice nice nice nice”; “I love getting on Twitter / Instagram and seeing another friend, loved, or/and mutual receiving their vaccine. Just makes me so insanely happy" and "Got the vaccine! So thankful for God’s protection all around me!”*.
  These posts demonstrate positive public perceptions based on high levels of confidence that vast segments of the population have in vaccines. There is also a strong positive public perception on the operations and delivery of the vaccines: *“Super shout out to everyone at the Moorestown Mall COVID-19 vaccine mega site. Unbelievably efficient & well run....really impressive...”; “Today I got my second dose of the Pfizer COVID-19 vaccine. Kudos to the Vermont National Guard for a job well done: well organized, smooth, courteous.”; “Best ‘day-date’ ever with @john_r_ratliff Our 1st vaccine dose is DONE. Thank you @UofLHealth for the massive undertaking, exceptional organization, and seamless process. #pfizercovid19vacccine #SeeYouIn3Weeks”* and *“Got my first COVID vaccine this morning administered by @NashvilleHealth. They are doing a phenomenal job with the logistics of rolling this out to our city. Everyone was friendly, helpful and efficient. Big thanks and kudos to all involved!!”*.
  These posts demonstrate public sentiment being driven by the vaccines and by their smooth delivery. An important takeaway from this for public policy is the emphasis on facilitating a comfortable and efficient vaccination process.
(b) **Scenario 2: Positive public sentiment decreases from early February to late March 2021.** Interestingly, we saw a decrease in positive public sentiment as indicated by the change in the proportion of positive tweets from early February to late March 2021. We do not speculate on the reasons for this but, instead, attempt to identify and discuss mass perceptions to help the public policy discourse.
  Skepticism, both reasonable and unreasonable, appears to have increased, which may have toned some proportion of positive tweets into neutral or negative territory: *“@Ironman_E @cruddydre_ @Ms_Jaydee But if your vaccinated and the vaccine works, what does it matter if im vaccinated? Youre safe right? Because the vaccine works right?”; “If we’re supposed to get the vaccine to protect other people but other people are supposed to get the vaccine to protect other people, who is it actually protecting? If the vaccine is already protecting those other people how does other people getting it further protect them?"; "excited to announce that i am not 30, therefore ineligible to receive a vaccine in the state of New York” and “@7_bunnies @TwixxBar07 @courtneymilan “Experts do not know how long” I’m protected by the antibodies I’ve acquired from infection. That’s fine. They don’t know how long the vaccine will protect, either, so we’ll all learn together. Meanwhile, I’ll stick to my own immune system’s protection”*.
  This change in sentiment, though minor, may be mitigated by a number of factors, such as a general loss of enthusiasm without a loss of confidence in the vaccines. Public policy can be quite useful in addressing such scenarios to prevent the erosion of positive public perceptions of vaccines and on the vaccination process—this can be achieved through appropriate communications and pronounced narrative development.
(c) **Scenario 3: Negative public sentiment increases from early February to late March 2021.** Multiple sentiment scoring methods affirmed the proportional increase in negative sentiment from early February to late March 2021. This is more alarming than the minor loss of positive sentiment across the same period: a loss of positive sentiment can be attributed to a number of factors and may not necessarily reflect a loss of confidence in vaccines; however, a proportional increase in negative sentiment over time could imply eroding confidence in vaccines based on rumors or inaccurate information. There could also be statistically disproportionate emphasis on some cases of people falling ill after taking the first or both dose/s of the vaccines, or outlier events, such as deaths following vaccination.
  Illustrative tweets convey these concerns and negative sentiment quite effectively: *“Whoa...when they say that the second vaccine might have strange side effects, the are NOT kidding.”; “Vaccine update: a day after shot one of the Pfizer vaccine I’m definitely feeling the arm pain, a headache, and kind of run down.”; “@onewiththesand Oh no. Feel better. Do you feel it is from your vaccine shot?” and “I would not have taken the vaccine if I knew it would make me sick... ”*. While there is a general concern about some uneasiness, discomfort and pain post-vaccination, most people appear to accommodate that, as illustrated by many tweets along the lines of: *“pretty achy and fatigued following my second vaccine dose yesterday. but still so grateful and hopeful. worth it 1000x over! #GetVaccinated ”*.
  Perhaps, the greater challenge is posed by some factual news on statistical outliers augmented by rumors of people being severely affected by vaccinations or even dying from the vaccine. This is illustrated by the deeply negative sentiment in some of the vaccine tweets in March of 2021: *“@cruddydre_ My uncle got the vaccine and died a week or 2 later. I’m good bro yal can have that vaccine.”; “@DanMac2014 @Kayla_Grey @pfizer Wish one could request your vaccine, @moderna was a monster. Haveing had COVID recovered in 3 days with light fever/aches. Vaccine-1st dose F’d me up-5x worse then VID did. I wanted to crawl under a rock & die the past 3 days. Not sure I’ll go for 2nd...”; “Mom don’t be a cry baby. It doesn’t help that I have a sister that tried to have her not take it telling her that there’s people who died from the vaccine...I tell my mom that my sister isn’t well informed...directs mother to the daughter who has a PhD.”; “@princessnofrog Haven’t seen that yet they are not sure because it’s still in the experimental stage but there has been people that have died from the vaccine. Meaning it can be deadly” and “Maybe the vaccine will kill me since the virus didn’t”*.
  Such expressions of fear, despair and negativity tend to spread fast through social media and personal messaging and communications. Public policy is critical in such scenarios and proactive steps are required to build public confidence. Transparency on adverse events must not be compromised—post-vaccination illnesses and the rare cases of death must continue to be documented and such information must be made publicly available.
  However, public policy must mandate resources for such information to be augmented and qualified by factual information truthfully highlighting the details along at least two lines of reasoning: (a) An emphasis on the rarity of extreme events such as vaccination induced deaths and (b) disassociation of vaccination as the cause of deaths after vaccination, where applicable, and clarity on the identified or potential causes of death.

### 4.2. Word Frequency and N-Gram Analysis

**Figure 4:**
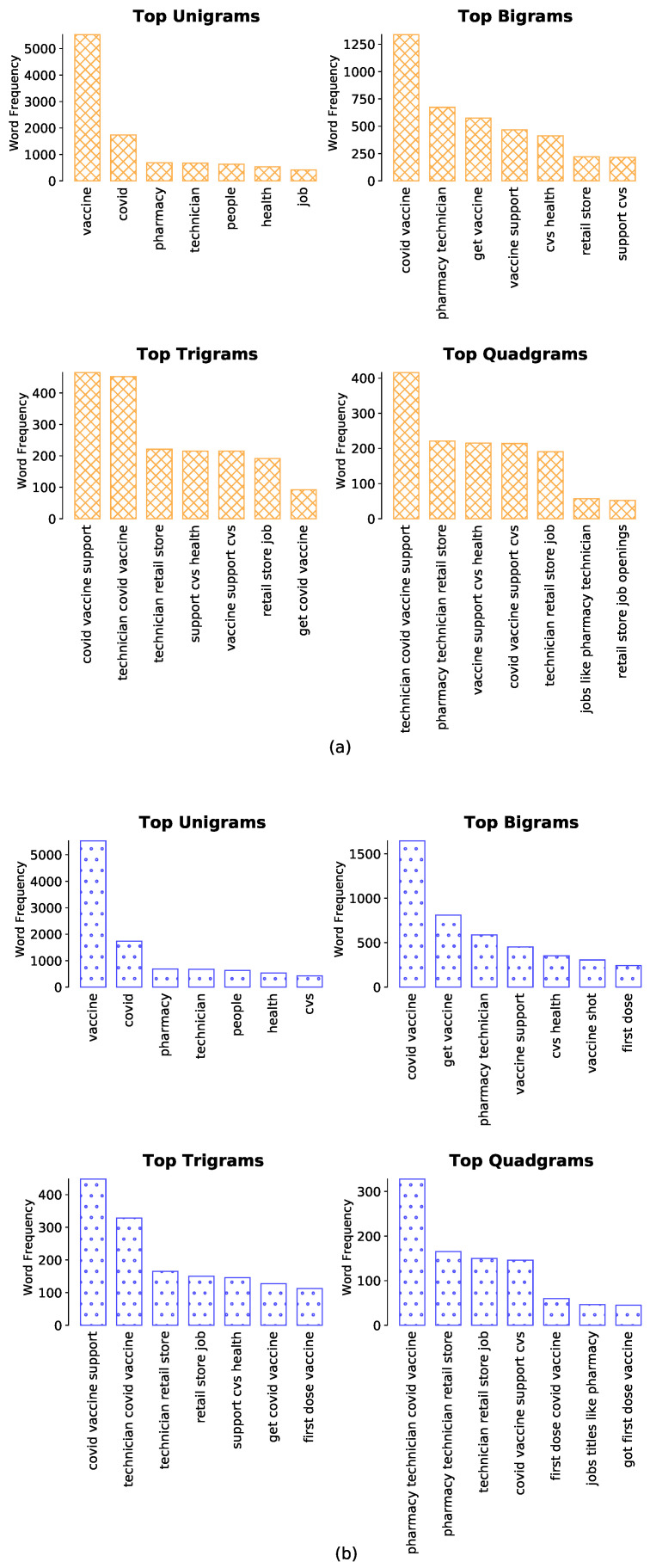
N-grams. (**a**) Tweets posted in early February 2021. (**b**) Tweets posted in late March 2021.

Word frequency and N-gram analyses are shown in Figure 4 (Figure 4a with a crossed pattern for February tweets and Figure 4b with a dotted pattern for March tweets). Word frequency analysis provides an array of high-frequency words, sorted by decreasing frequency: *“vaccine, covid, pharmacy, technician, people, health, job”*. Many of the high-frequency words were observed to be in tweets supporting positive sentiment towards vaccination.

The top Bigrams (two-word sequences) included: *“covid vaccine, pharmacy technician, get vaccine, vaccine support, technician covid, cvs health”*, and the Bigrams also indicated frequent usage and support for positive sentiment on vaccination. The top Trigrams (three-word sequences) were more insightful about the mass focus on vaccine distribution and operations. They were mostly aligned with support for positive sentiment about vaccination. The Quadgrams also showed reasonable support for positive sentiment about vaccines, and emphasized the operations and delivery aspects of the vaccine. Lower frequency Quadgrams provided insights into emerging themes or common concerns, including concerns whether governments were arranging sufficient vaccine supplies to be distributed and related challenges.

### 4.3. Temporal Changes in Public Sentiments

**Figure 5:**
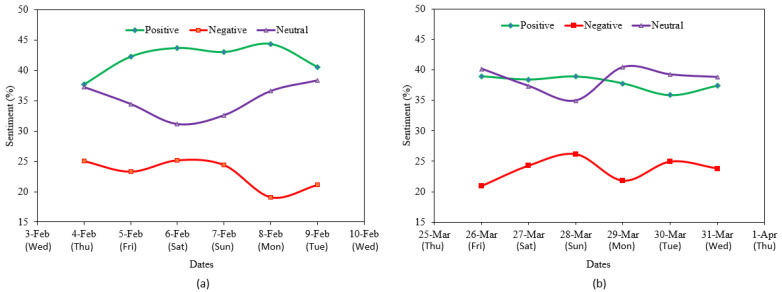
Temporal sentiment towards vaccines from tweets extracted in (**a**) the first week of February 2021 and (**b**) the last week of March 2021.

COVID-19 vaccine sentiments among Twitter users were observed and analyzed over a period of 6 days, both in the early February and the late March 2021 time frames. In the February time frame, an increasing positive sentiment trend, a decreasing negative sentiment trend, and stable neutral sentiment trend were found (Figure 5a). On the other hand, in the March time frame, at the end of the week, no significant change was observed in the positive and neutral sentiment trends, but a slight increase is discernible in the negative sentiment trend (Figure 5b). Apart from this, we also noted that the negative sentiment score was the lowest on Monday in both time frames. Negative scores were higher over the weekends compared to weekdays. Conversely, the neutral sentiment score was the lowest during the weekends. This suggests that people post more negative tweets during the weekends than on weekdays.

**Figure 6:**
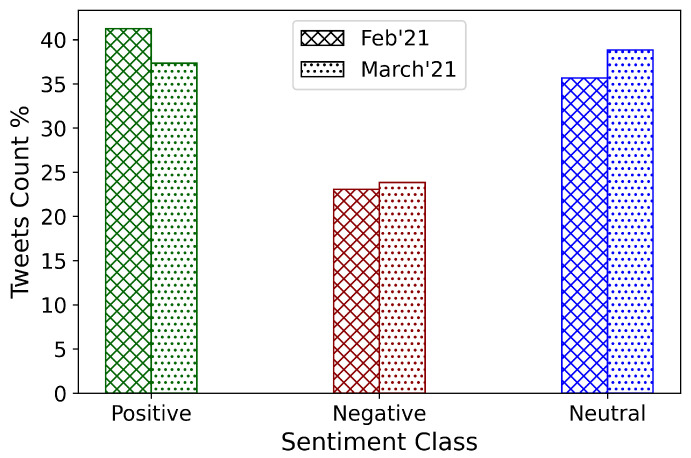
Change in the overall sentiment scores from early February to late March 2021.

Overall, as depicted in Figure 6, the share of positive sentiment tweets decreased over time (from early February to late March 2021). On the other hand, although the ratio of negative sentiment tweets did not change much, neutral sentiment increased noticeably. This indicates that, while COVID-19 vaccination was in full swing during February and March of 2021, public perceptions towards vaccination did not improve on the aggregate.

**Figure 7:**
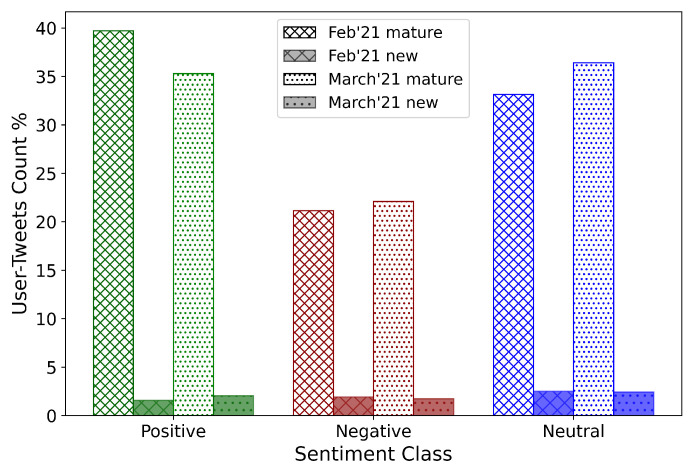
Temporal sentiment analysis in early February and late March 2021 by the type of Twitter account users (new users vs. mature users).

To understand the effect that the type of Twitter users may have had on the sentiment score, the Twitter accounts were divided into two groups based on the year of creation of the account. Accounts created before January 2020 form the mature group, while those created subsequently were categorized as the new group. The tweet sentiments based on these age groups were observed and plotted in Figure 7. Of the total number of tweets, 6% and 6.21% were posted by new users in early February and late March 2021, respectively. These results indicate that tweets by new users neither dominated the overall narrative on COVID-19 vaccines nor, therefore, the public sentiments. Among the February tweets, about 40% were mature positive, 21.15% were mature negative, and 33.15% were mature neutral tweets.

On the other hand, in March 2021, 35.30% were mature positive, 22.09% were mature negative, and 36.40% were mature neutral tweets. It can be observed that, both among February and March tweets, positive sentiment was higher than negative sentiment among the mature users. Additionally, the percentage of neutral sentiment increased among the mature users from February to March. However, no such discernible change can be observed in the posts made by the new users. Sentiment changes in the Twitter discourse are entirely imputable to mature users.

### 4.4. Statistical Analysis of Sentiment Values

Various characteristics of the tweets were explored using descriptive analytics, and tweet length was found to have no significant correlation to sentiment scores (Figure 2). Tweet lengths and sentiment scores displayed bimodal and skewed visually undetermined distributions. The majority of tweets were between 10 and 55 words in length, and most of the tweet sentiment scores fall in the range of −1 to 1. Our analysis of the sentiment scores shows that the percentage of tweets with positive sentiment was higher (daily as well as overall, in both datasets) compared to the percentage of tweets with negative sentiment (Figure 5).

**Figure 8:**
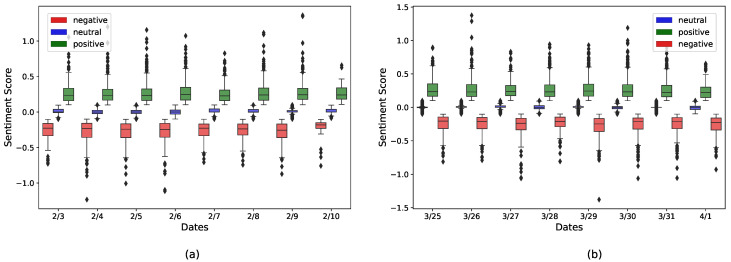
Boxplots for the sentiment scores. (**a**) February 2021 sentiment scores. (**b**) March 2021 sentiment scores.

Figure 8 shows day-wise boxplots representing the distribution of sentiment scores in three classes (positive, negative, and neutral) based on the February dataset (Figure 8a), and March dataset (Figure 8b). It can be observed that the median values of the positive sentiment scores were equal or slightly greater than 0.25, whereas the maximum values were usually over 0.50. On the other hand, the median values for the negative sentiment scores were close to −0.25, and minimum values were usually under −0.50.

For both positive and negative sentiments, the median value was not located in the middle of IQR (interquartile range) of the boxplot, which implies that the sentiment scores were most likely not normally distributed. Additionally, we observe more outliers in positive sentiment boxplots than in negative sentiment boxplots (more tweets in extremely positive class than extremely negative). Descriptively, these boxplots indicate that the overall strength of positive sentiment was greater than that of the negative sentiment.

**Figure 9:**
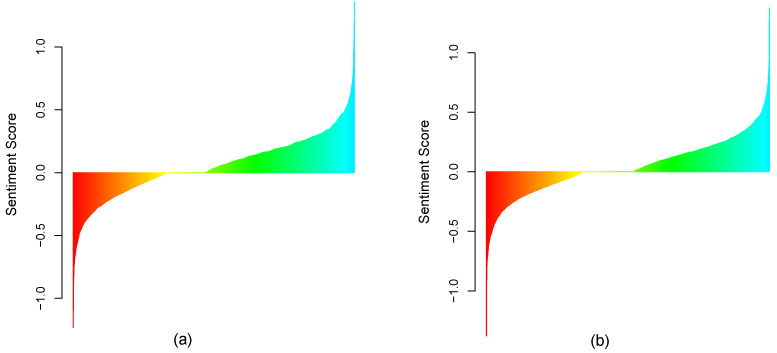
Sorted sentiment score barplots (**a**) February 2021. (**b**) March 2021.

The descriptive analytics presented thus far provides strong support for a positive sentiment on vaccines that dominates the corresponding negative sentiment for both February and March 2021 (see Figure 5 in particular). There is also some initial evidence of a decrease in positive sentiment between February and March, and evidence of an increase in negative sentiment between these time frames. The visual analysis of sorted sentiment scores for early February and late March 2021 (Figure 9a,b) suggests the same.

However, this is insufficient from a statistical standpoint, and it is necessary to validate such descriptive findings. We validated the exploratory findings with a Proportion test on the ratio of positive tweets to negative tweets separately for early February and for late March, and further verified this by applying an Exact Binomial test as shown in Table 2, Table 3 and Table 4.

#### 4.4.1. Dominant Positive Sentiment for February and March 2021

**Figure 10:**
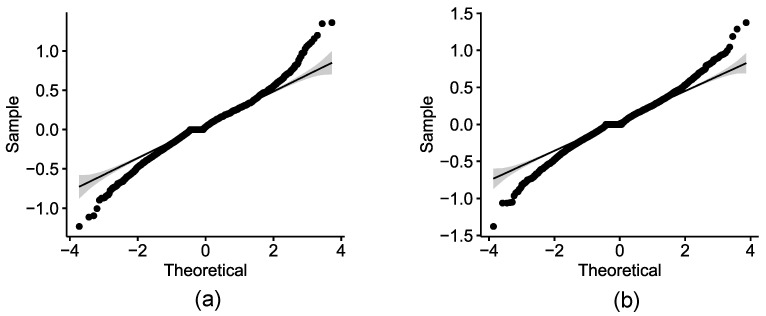
Q-Q plots. (**a**) February sample. (**b**) March sample.

We first tested for the normal distribution of the sentiment scores. For this purpose, we applied the Shapiro–Wilk normality test on the sentiment scores computed for February 2021 (Table 2) and for March 2021 (Table 3). The tests concluded that the sentiment scores were not normally distributed. These results were also supported by the Q-Q plots indicating departure from normality (Figure 10). We therefore employed the same validation approach used for the PSS framework by Samuel et al., which involved applying the non-parametric Proportion Test and Exact Binomial Test [5].

The results of the Proportion Test, with the null hypothesis indicating that the proportional count of negative tweets is equal to or greater than that of positive tweets, inferred that the null hypothesis could safely be rejected with a *p*-Value significantly <0.0001 on both the February and March samples. The Proportion Test results, thus, validated the alternative hypothesis for the positive sentiment proportion being significantly greater than the corresponding negative sentiment. The Exact Binomial tests confirmed the same results.

Finally, the Wilcoxon signed rank test was used to confirm the findings, as the sentiment scores were not normally distributed. Thus, the dominance of the positive sentiment scores for both February and March were supported both by the Proportion Test, as well as by the Exact Binomial Test.

#### 4.4.2. February-March: Lower Positivity and Higher Negativity

Although, as demonstrated above, positive sentiment for both February and March remained stronger than corresponding negative sentiment, our analysis of relative changes highlighted important insights: the relative proportion of positive sentiment decreased from February to March, and the relative proportion of negative sentiment increased from February to March 2021. Here again, we employed the Proportion Test and further validated it using the Exact Binomial Test. Our null hypothesis was that positive sentiment remained in the same relative proportion or increased from February to March.

The results, as shown in Table 4, indicate that the null hypothesis can be fairly rejected in favor of the alternate hypothesis that relative proportion of positive sentiment decreased from February to March. In a similar way, we statistically tested that the relative proportion of negative sentiment increased from February to March. The original PSS framework applied these methods in a cross sectional manner, with data collected for one period of time [5]. In this study, we extended the PSS framework to contrast Public Sentiment Scenarios across two time periods, namely February and March 2021.

### 4.5. State-Wise Sentiment Analysis in the US

In this subsection, we explore the progression in COVID-19 vaccination sentiment on a state by state basis from early February to late March 2021. It should be noted that, because of the limitations of geo-location of Twitter posts, some states may have rather small sample sizes, and therefore inference should proceed with caution. This is particularly the case of Idaho, South Dakota, Vermont, and Wyoming in February and of Alaska and Wyoming in March.

**Figure 11:**
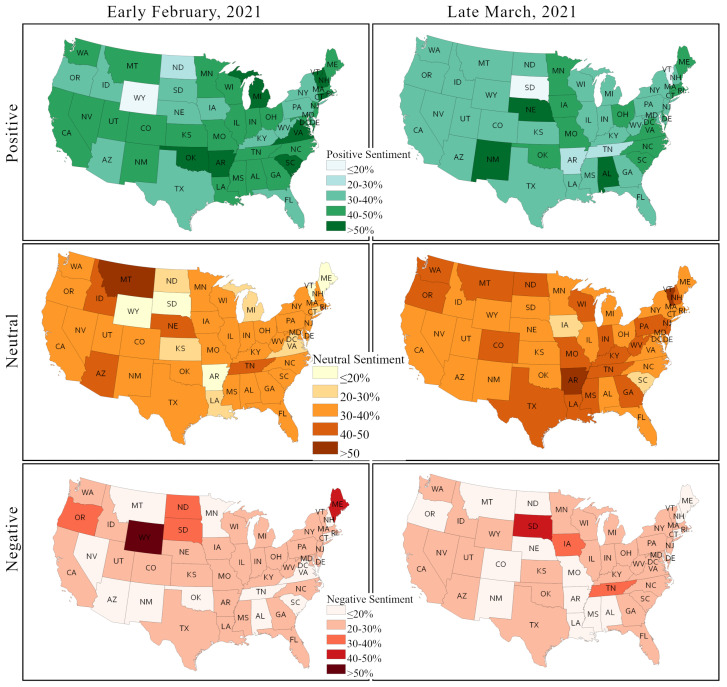
Changes in positive, neutral, and negative sentiment in US states.

Figure 11 depicts the percentage of positive, negative, and neutral sentiment across states in early February and in late March. The positive sentiment is decreased in most states, except for a handful, namely New Mexico, Nebraska, Iowa, North Dakota, and Alabama. In the latter states, the positive sentiment is increased by as much as 10%. The largest decline in positive sentiment is observed in Vermont and Arkansas, from more than 50% to below 20% and 20–30%, respectively. A few states have around 20% decline in positive sentiment including New Hampshire, Michigan, South Dakota, and Tennessee, whereas South Dakota and Tennessee have a significant increase in negative sentiment. Most of the states have no or very little change in negative sentiments.

A significant decline in negative sentiments is observed in only four states: Wyoming, North Dakota, Oregon, and Maine. A significant decline in both positive and negative sentiments in Arkansas results in a sharp rise of neutral sentiments. In contrast, both positive and negative sentiments are increased in a few states, while neutral sentiment is decreased, as in the case of Iowa.

**Figure 12:**
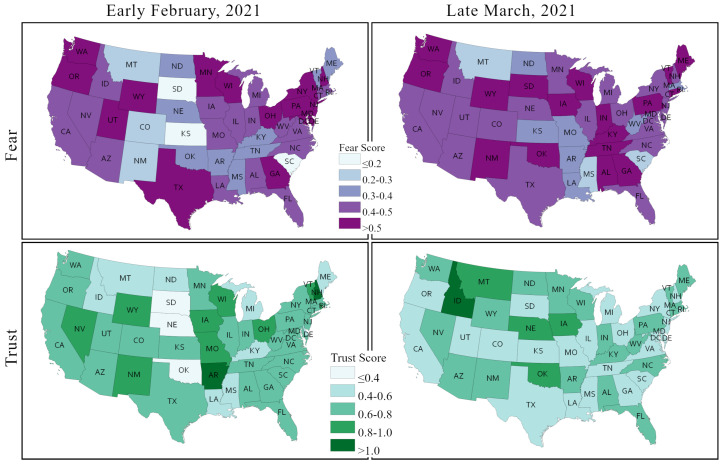
Changes in fear and trust sentiments in US states.

Figure 12 depicts the changes in two sentiments that feature prominently in the context of the COVID-19 pandemic and of the health policy responses, including the vaccination campaign, –fear and trust– between early February and late March across US states. The fear sentiment vis à vis COVID-19 vaccination is very strong in most states, and this sentiment gained even further strength in most cases over time. Nearly two thirds of the contiguous US states showed an overall fear score over 0.4 in early February. This number increased to 38 (about 80%) by late March. Generally, the fear sentiment remained unchanged in the Northwest and the West regions, but it increased in many states of the Southwest, the Southeast, and the Midwest. Fear sentiments decreased or remained unchanged in several large states, including Texas, New York, California, Arizona, and Florida.

Most of the states showed relatively moderate to low score in trust sentiments in early February. However, in contrast to the fear sentiment, the trust sentiment dropped further in a majority of states, except for a few, such as South Dakota, Nebraska, Oklahoma, Idaho, Arkansas, and North Dakota. Trust in vaccines decreased the most in Arkansas. Although, fear sentiment remained largely unchanged in large states, trust sentiment decreased in California, Texas, and New York.

### 4.6. Discussion

Considering the criticality of vaccination to secure public health and the high ambition of the stated national goal, we sought to identify the states that could be regarded as leaders and laggers in the pursuit of vaccination, and to assess how the differentiation among states on this basis intersected with state-wise public sentiments. To this end, we used the state-wise actual rate of vaccinations sourced from the US CDC [12]. We observed that the vaccination rates varied considerably from one state to another. We will discuss state-wise sentiment assessed from the Twitter posts in relation to these vaccination rates and vice versa.

**Figure 13:**
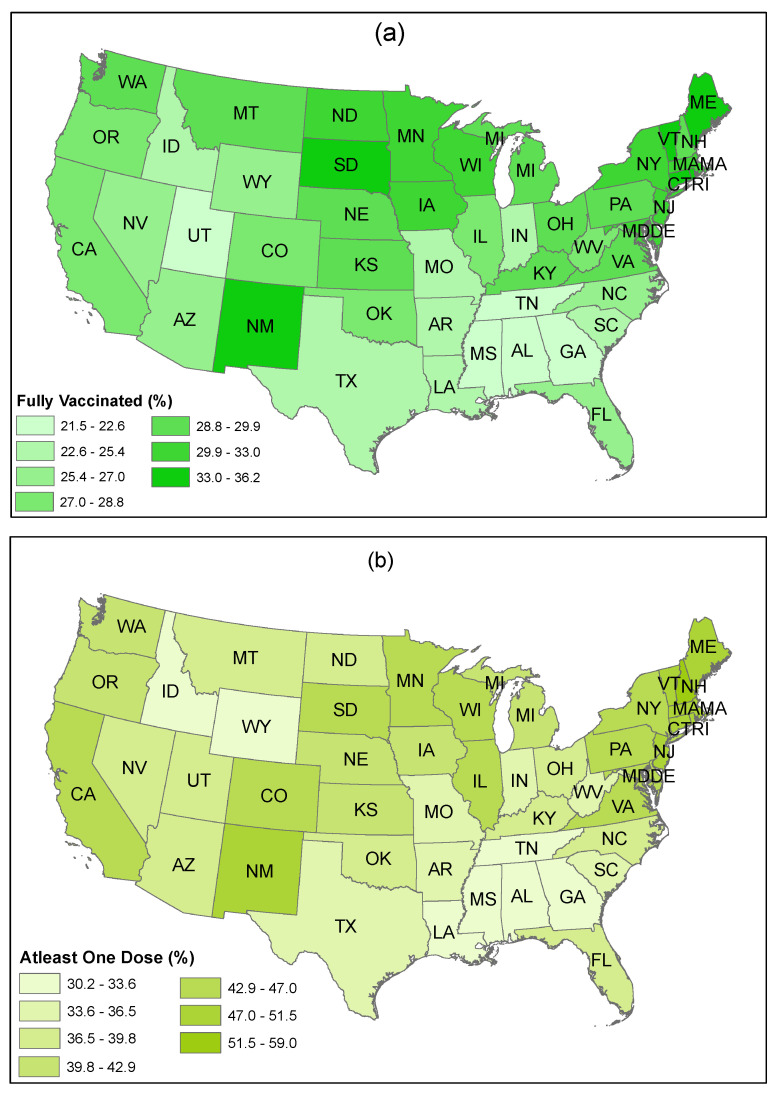
(**a**) Fully vaccinated people per 100 persons; (**b**) People with at least one dose of vaccine per 100 persons (Data source: [12]).

Figure 13 shows the rate of fully vaccinated people and of people who have received at least one dose of vaccine (%) in US states as of 28 April. The figure indicates that people living in the Northeast (e.g., Maine, New Hampshire, Connecticut, Rhode Island, Vermont, New Jersey, Massachusetts, New York, Pennsylvania, Delaware, the District of Columbia, and Maryland) have a higher rate of vaccine uptake. In contrast, a lower rate of vaccine uptake is observed in the South (e.g., Alabama, Georgia, Louisiana, Mississippi, Tennessee, Arkansas, South Carolina, and Texas).

A somewhat mixed response to vaccination can be noticed among the residents in the Midwest and in the West (i.e., some states have a higher vaccination rate, while some have a lower rate). People living in the West, particularly in California, New Mexico, Arizona, and Colorado showed a higher willingness accompanied with a higher rate of vaccination. However, residents from Wyoming and Idaho showed a reluctance to accept vaccines, and, as a consequence, a lower rate of vaccination is observed in these two states.

Although a higher use of distributed vaccines is observed in Utah, overall vaccine administration is low in this state. The states in the Midwest also have a higher rate of vaccine uptake. More specifically, people in South Dakota, North Dakota, Wisconsin, Iowa, Kansas, and Minnesota have a higher rate of vaccination. On the other hand, Missouri and Indiana have a lower rate of overall vaccine administration.

**Figure 14:**
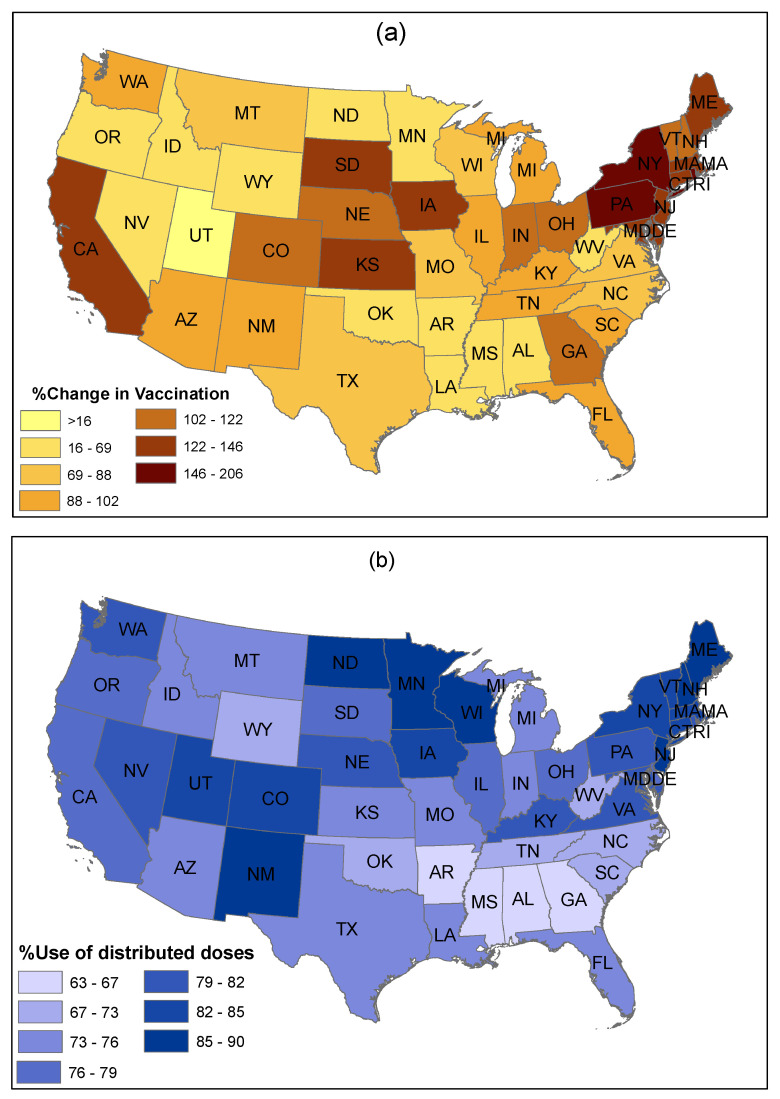
(**a**) Relative changes in vaccination between February (average vaccination from 3 to 10 February) and March (25 March to 1 April), 2021, (**b**) Percentage use of distributed vaccines, as of 28 April (Data source: [12]).

To more directly compare state-wise vaccination propensities with our tweet sentiment analytics, we also investigated the changes in vaccination uptake in the US by collecting average daily vaccination data from the same time frames, that is 3 to 10 February and 25 March to 1 April (Figure 14a). The figure shows that most states achieved a significant increase in vaccination in late March, when compared to early February. The highest increase in vaccination is observed in the Northeast regions of the US.

Similarly, a significant increase in vaccination is also observed in some Midwestern states (e.g., Kansas, Iowa, and South Dakota), despite a low increase in vaccination in North Dakota and Minnesota. In contrast, the lowest increase in vaccination is noticed in parts of the West region (e.g., Utah, Idaho, and Wyoming). However, California achieved a higher increase in vaccination from early February to late March. Finally, many Southern states (e.g., Mississippi, Louisiana, Alabama, Oklahoma, Arkansas, and West Virginia) achieved a relatively low increase in vaccination between the two time frames. Sparsely populated states and less populous states achieved a higher rate of vaccination in comparison to more populated states.

Extant studies have mentioned that one of the main reasons for a low acceptance of vaccines is the inadequate supply and distribution of vaccines and low accessibility to vaccines. Considering this issue, we investigated the share of vaccine doses used from the actual distribution in different states of the US to gauge the status of vaccine dose utilization, as of 28 April (Figure 14b). This figure shows a comparative scenario of differences between vaccine used and vaccine distributed across states.

This evidence verifies that Northeastern states have a higher percent usage of the distributed vaccines. Similarly, a significant use of distributed vaccine is observed in Western and Midwestern states. In contrast, Southern states showed a lower percent use of the distributed vaccine, which is consistent with the lower rate of vaccination uptake in these states as shown in Figure 13.

To understand the root causes of vaccine hesitancy among Americans, a recent nationwide Household Pulse Survey (HPS) conducted by the US Census Bureau (from 14 April to 26 April 2021) investigated the status of COVID-19 vaccine hesitancy at the county level of each state [13]. Survey participants were asked the following question: “Once a vaccine to prevent COVID-19 is available to you, would you get a vaccine?” with following four options proposed as answers: (1) “definitely get a vaccine”, (2) “probably get a vaccine”, (3) “probably not get a vaccine”, and (4) “definitely not get a vaccine”.

**Figure 15:**
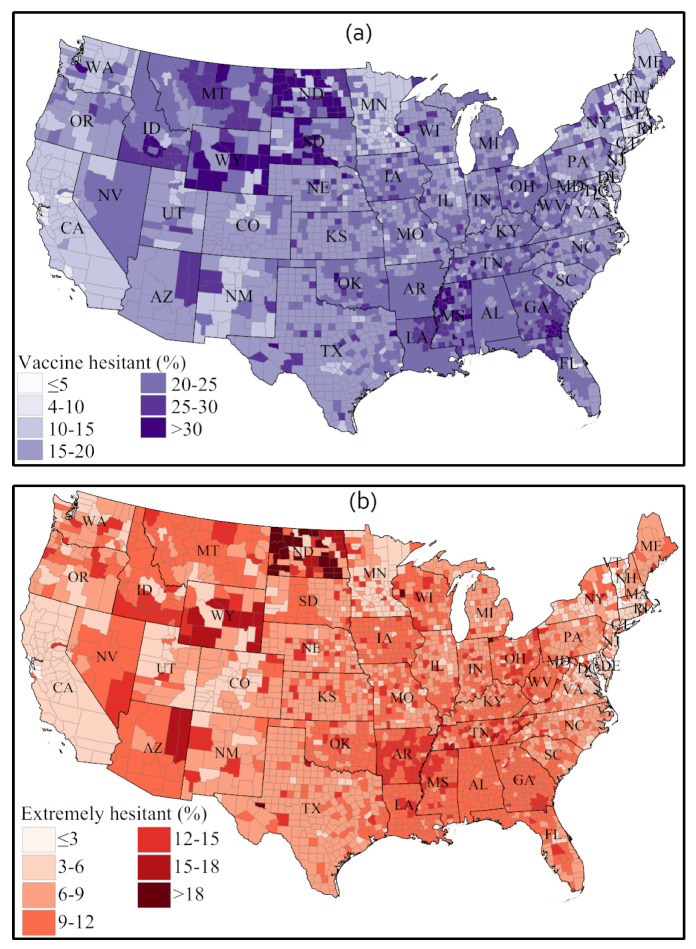
(**a**) COVID-19 vaccine hesitancy (%) in US counties; (**b**) COVID-19 vaccine extreme hesitancy (%) in US counties (Data source: [13]).

The survey took the “probably not” and “definitely not” responses for vaccine hesitancy, while the “definitely not” option is defined as extreme vaccine hesitancy. The spatial representation of vaccine hesitancy in Figure 15 indicates that many counties in Western states, parts of the Midwest, and parts of the South have a higher incidence of vaccine hesitancy and extreme vaccine hesitancy. Consistently with these survey results, a lower rate of vaccination was observed in most of these states.

Table 5 shows the prominent reasons for COVID-19 vaccine hesitancy reported in the HPS survey [13]. The possible side effects of vaccines is reported as the main reason nationally (20.86%), whereas safety (17.04%) and trust (12.55%) of vaccines are the other two important reasons why Americans are hesitant to take the COVID-19 vaccine. Almost 10% of respondents also said they declined vaccination due to a lack of trust in the government.

In another study, the YouGov market research and data analytics firm, in association with the Institute of Global Health Innovation (IGHI) at Imperial College London (ICL), conducted surveys in 29 countries to gather global insights on people’s behaviors in response to COVID-19 [66]. Respondents, who did not receive a COVID-19 vaccine, were asked to provide their responses on a scale of 1 (i.e., strongly agree) to 5 (i.e., strongly disagree) to the question: “If a COVID-19 vaccine were made available to me this week, I would definitely get it”.

Only 34.5% of US respondents expressed their willingness to receive a COVID-19 vaccine, which validates our findings from Twitter sentiment analysis (i.e., roughly 37% of US Twitter users expressed positive sentiment on the COVID-19 vaccine (Figure 6)). As a point of comparison, in this international survey, UK residents had the highest positive responses overall (67.2%), followed by residents of Denmark (66.8%), Norway (58.5%), Germany (58%), and Italy (57.3%).

From our PSS-framework-based sentiment analysis of Twitter posts over two time periods in early 2021 (Figure 11 and Figure 12) and from the US state-wise actual vaccination scenarios over the same periods (Figure 13, Figure 14 and Figure 15), we identified significant changes in sentiment from February to March and found that both the public sentiment and vaccination administration scenarios varied from one state to another state and have a strong correlation with population density and agglomeration, and with geographic location. Some of the notable key points are stated below.

1. Sentiments towards vaccination vary considerably across states and other place-based communities, like counties, and so do vaccination rates and hesitancy. We observed that less populous states and more sparsely populated states tend to have a higher vaccination rate; some of these states also have a strongly positive vaccination sentiment, but some also exhibit high vaccination hesitancy and less positive sentiments.
2. The Northeast region has a higher vaccination acceptance than other regions of the US. The strongest vaccine hesitancy is found across a number of southern states, such as Alabama, Georgia, Mississippi, Tennessee, and Arkansas. The Midwest and the West show mixed vaccine acceptance scenarios. Notably, Wyoming and Idaho show lower vaccine acceptance. In contrast, California, New Mexico, Arizona, and Colorado show a higher vaccination acceptance. The HPS survey found that side effects, trust in vaccine and in authority, safety, and efficiency are the main reasons for lower vaccination acceptance in these states.
3. Although positive sentiment declined and neutral sentiment increased in most states from February to March 2021, we found that most states improved their actual vaccine administration rate. We argued that this can be imputed to the increasing state-wise vaccine availability as time progresses.
4. Among the specific negative sentiments isolated from the Twitter corpus (including fear, which was discussed explicitly, but also sadness, anger, and disgust), fear is the sentiment that strengthened the most over time in most states.
5. The fear sentiment remained unchanged (between early February and late March) in populous states, like California, Texas, and New York), whereas a slight decline in trust sentiment was observed in these states.
6. Changes in sentiments were very abrupt among less populous states in the central parts of the US, including Wyoming, South Dakota, Kansas, New Mexico, and Arkansas.

In summary, as vaccine hesitancy exhibits significant variability from one state to another, state-wise policies need to be sensitive to these variations instead of adopting a “one size fits all” approach to increase the vaccination uptake. In Section 5, we apply the PSS framework logic to propose public policies that could be adopted by local, state and federal governments to provide increased sensitivity to the masses and responsiveness to public sentiment, in the pursuit of herd immunity.

### 4.7. Limitations

The current study significantly contributes to the scientific knowledge base by investigating public perceptions towards the COVID-19 vaccines in the US amid widespread misinformation on vaccines, lack of trust in vaccine and in authority, and vaccine hesitancy. Despite the timely contributions made to the literature, this study also has certain limitations, which are stated below.

- First, although researchers and policymakers have used Twitter data quite widely, Twitter posts may inadequately represents perceptions and opinions of the people from certain strata of society [18,67]. Many people (e.g., low-income and low-education people, and the elderly) do not have access to Twitter or shy away from social media, and therefore Twitter data may inaccurately characterize the sentiment towards vaccination in the population.
- Second, Twitter data are susceptible to bot activities [5]. Twitter bots are programmed to mimic daily Twitter users and share contents on a specific subject matter to spread news to the broader audience, which could be hoaxes and fakes and could alter the perceptions of actual Twitter users [68]. Moreover, voluntary inaccuracies and involuntary mistakes by users produce inaccurate data. Thus, the quality of Twitter data is reasonably affected by bot activities and user mistakes.
- Third, the sentiment scores extracted from Twitter posts using the Syuzhet package have some degree of inaccuracy for sentiment scores assigned to individual tweets, which may influence sentiment analysis. Such errors in sentiment scores are common across libraries and packages for textual and sentiment analytics, and the general expectation is that the overall sentiment scores remain fairly accurate subject to availability of sufficient data. Considering this issue, we used two more lexicon-based python libraries (e.g., Textblob and VADER) to validate the sentiment scores estimated using the Syuzhet package. However, future studies should consider fusing information from Twitter messages and sentiment propagation patterns to achieve better performance in analyzing Twitter data [69].
- Fourth, while our study demonstrated the unique value of Twitter posts to probe the sentiment of populations towards a current issue, such as vaccination, our work is preliminary, being based on two time frames only and on geo-location information that constrains the size of samples at a desirable spatial granularity, i.e., the state, the city, or the county. This work calls for further spatial-temporal monitoring at fine granularity over the long term.
- Fifth, this study explored the public sentiment towards the COVID-19 vaccines in the US and investigated the spatial and temporal changes in the sentiments. However, a study evaluating the key reasons for positive and negative sentiments would be desirable for targeted interventions of public agencies to increase vaccine uptake.

## 5. Policy Implications

Based on the sentiment analysis we conducted and on our review of multiple surveys, we posit that local, state and federal governments can leverage our PSS framework driven analysis to influence and shape policy initiatives for improving COVID-19 vaccination rates. Our analysis studied sentiment changes at the national and state levels, and our corresponding potential policy implications are stated below.

- First, there is a significant opportunity for governments to improve vaccine distribution and delivery and perceptions surrounding these tasks. Our Twitter sentiment analysis and our review of associated information and multiple related surveys, demonstrated that the vaccine distribution and delivery process is one of the prominent reasons why people are unhappy or uncomfortable with vaccination. Therefore, federal and state governments should adapt their policies to both improve the distribution system and process, and also improve communications about the distribution and delivery process to address public concerns.
- Second, from the temporal sentiment analytics studying public sentiment changes from early February to late March 2021, we observed that simply giving more time did not help to improve public perceptions of COVID-19 vaccination. When vaccines became available to an increasingly large population in the US, it did not proportionately boost the vaccination rate [70]. Hence, the findings of our study imply that it would be beneficial for federal and state governments to take proactive actions to specifically attract vaccine hesitant segments of the population to be vaccinated. For example, the state of West Virginia offered $100 savings bonds to 16–35 years vaccinated adults. The state of Maryland offered $100 to all vaccinated state employees. In Connecticut, New Jersey, and Washington D.C., there are free-drink offers for vaccine takers [71]. An investment in such initiatives has the potential to break barriers and increase the COVID-19 vaccination rates by great margins.
- Third, from our application of the PSS framework and our thematic review of tweets, it is evident that people have hesitation and fear about the side effects of vaccination and have doubts about the effectiveness of the vaccines, which led to increasingly negative sentiment from early February to late March 2021. This scenario creates a need for governments to launch nationwide and statewide public awareness programs to educate and better inform people about vaccines, their side-effects, and effectiveness in a transparent and professional manner.
  It may be possible to learn from states where public sentiments show higher proportions of joy, trust and anticipation towards vaccines. Exceptional success stories need to be identified and disseminated through mass media to create a positive narrative and strengthen the positive sentiment towards vaccination. Moreover, sentiment analysis from this research can be associated with socioeconomic factors for further research to know how culture, income level, education level, and religious beliefs affect sentiment and public opinion variance towards COVID-19 vaccines.
- Fourth, some studies (e.g., [32]) observed a higher vaccine hesitancy among specific segments of society (e.g., Black/Hispanic population, less educated individuals, and rural residents). This appears to be supported by linguistic patterns in certain tweets with negative sentiment. Thus, targeted and multi-faceted efforts could be effective at increasing the acceptance of COVID-19 vaccines by these segments of society.
- Fifth, an important insight from our application of sentiment analytics and textual thematic reviews is that some segments of the population are hesitant to take COVID-19 vaccines because of a lack of trust based on the knowledge of the fast-tracked development and approval of the COVID-19 vaccine, whereas other vaccines, like Measles or Mumps, historically took significantly longer times to develop, test and approve and are, therefore, perceived to be safer and more effective.
  Governments and vaccine manufacturing companies need to proactively develop policies to publicize the reasons and the science behind the relatively rapid development and emergency approval of the use of COVID-19 vaccines via social media and supporting forms of mass communications. This could increase positive sentiment and public trust towards the vaccines.
- Sixth, government and corporate policies regarding news media and social media geared towards publicizing the vaccination of senior government officials, civic and business leaders, and other public and influential figures in society (e.g., celebrities idolized by hesitant cohorts of society) could provide great role models that could positively influence and motivate people to accept the COVID-19 vaccine [36]. We identified positive opinions on this topic in our textual thematic analysis of public tweets.
- Seventh, our analysis highlights the importance of using the PSS framework for generating insights on the collective public sentiment towards COVID-19 vaccines at both the federal as well as at the state levels. We found diverse changes between the national and local levels of aggregate public sentiment as shown in Figure 6, Figure 9 and Figure 11. Furthermore, the thematic analysis indicates that some segments of the population that are vaccine hesitant have a high degree of concern for their health, as opposed to other vaccine hesitant segments that display little concern regarding COVID-19 health issues.
  We posit that governments must, therefore, accommodate state and local level needs of the people, and also proactively cater to the opportunity to develop policies that support all segments of the citizenry, even if vaccine hesitant, in helping them safeguard their health by boosting natural immunity and by using methods they are most willing to accept.
- Eighth, there are two dimensions to this research: The first is the temporal and limited to the United States. This refers to the US specific sentiment analysis and public policy discussions, which is valuable despite much of the US population being vaccinated at this time. However, the second dimension of this research has long-term implications for all countries that are looking for ways to improve public policy and ensure better alignment and mass messaging. This research demonstrates an effective approach to near-real-time tracking of changes in public sentiment towards vaccines, and potential public policy strategies, which can be extended to many nations. Therefore, all of the public policy implications discussed above possess value beyond the current time period for which the data is analyzed, and are applicable and extensible to many other nations that continue to battle against waves of COVID infections, and a stream of COVID variants.

## 6. Conclusions and Future Work

In this research, we analyzed public sentiment on COVID-19 vaccines in the US on the basis of Twitter data. The findings from Twitter data analytics were verified, validated, and intersected with actual vaccination data from the US CDC and the recent Nationwide Household Pulse Survey (HPS). We analyzed the progression of public sentiment from early February to late March 2021. We found positive sentiment to be dominant over negative sentiment in both February and March 2021. However, interestingly, in the sentiment progression from February to March, we noticed that positive sentiment declined and neutral sentiment increased, along with an increase in negative sentiment. We demonstrated these findings both with descriptive analytics and statistical analysis.

We used the PSS framework to structure our hypotheses for statistical analysis. Finally, the hypotheses were tested using the Proportion test and Exact Binomial test and guided by the Wilcoxon signed rank test, thus, demonstrating that our findings are non-trivial.

In our state-wise sentiment analysis, we found that sentiment scores differed from one state to another. We also noticed that state-wise positive sentiment scores had a strong correlation with the actual vaccination in that state. Based on our sentiment analysis and extensive review of vaccination rates and survey data, we observed that simply giving more time may not attract more Americans to be vaccinated. Rather, the agencies and organizations of local, state, and federal governments need to make proactive efforts and provide incentives to attract vaccine-hesitant Americans to accept COVID-19 vaccines. Finally, we provided valuable input for future public polices to increase vaccine uptake and help reach the greatly desired herd immunity goal.

The current study was conducted in a time sensitive environment with Twitter data to gauge public perceptions of the COVID-19 vaccine. However, future analysis of vaccine sentiment based on information collected from multiple sources (e.g., Facebook, LinkedIn, Reddit, Gab, various public online discussion boards, etc.) could cover a larger audience from different and multiple segments of the US population [35,72]. Future research can leverage the current study along with information from multiple social media platforms in diverse formats (e.g., text, blogs, images, comments, etc.) to develop a wide-ranging array of scenarios for sentiment analytics and, thus, expand the application of the PSS framework.

Spatio-temporal granularity of the analysis should also be increased to provide better monitoring tools to tailor COVID-19 vaccination policies and norms for “here” and “now”. Future research can also be extended to analyze the relationship between the changes in sentiments, the spread of the COVID-19 pandemic, and the emergence of new variants of the virus that are more virulent, using multiple information sources.

Considering the enormous computational capabilities of machine learning techniques to handle complex and multifaceted problems, future studies should apply machine learning algorithms to classify and label tweets and other social media posts with improved accuracy to, thus, better reflect real-world contexts [17,73,74]. This study employed the PSS framework and provides a strong basis for formalizing public sentiment driven influence on policy formulation and implementation.

This is very valuable and can lead to applied solutions particularly in conjunction with automated artificially intelligent information generation processes, which, for example, can be used to machine generate intelligent tweet responses and build positive consensus, clarify misinformation, and boost supportive sentiment for the vaccine [75,76,77].

## Figures and Tables

**Table 1:** Perceptions of the public towards COVID-19 vaccines.

Study	Context	Data Source	Vaccine Acceptance (%)	Vaccine Hesitant (%)	Neutral (%)
[3]	England	Online survey	34.3–55.8	-	-
[19]	Finland	Online survey	66.67	-	-
[23]	Review study	Reviewed 15 articles	86.1 (Student), 54.3–77.6 (general people)	-	-
[29]	USA	Twitter	57.65	42.35	-
[31]	USA	Online survey	62.2	14.8	23
[32]	USA	Cross-sectional survey	57.6	10.8	31.6
[33]	USA	Hypothetical scenarios	56	-	-
[34]	USA	Online survey	69	-	-
[35]	USA and UK	Facebook and Twitter	56 (USA), 58 (UK)	24 (USA), 22 (UK)	18 (USA), 17 (UK)
[36]	Canada	Twitter	84.55	15.45	-
[37]	Twitter Polls	Twitter	82.8	8	6.8

**Table 2:** Statistical tests for positive and negative sentiment counts for February 2021. (**a**) Proportion test for positive sentiment. (**b**) Shapiro–Wilk normality test for sentiment scores. (**c**) Wilcoxon signed rank test for the mean of the sentiment scores.

	Proportion Test	Exact Binomial Test
Null Hypothesis	−ve senti ≥ +ve senti	−ve senti ≥ +ve senti
Alt. Hypothesis	True *p* is less than 0.5	True *p* is less than 0.5
*p*-Value	2.20 × 10^−16^	2.20 × 10^−16^
Conclusion	Reject Null Hypothesis	Reject Null Hypothesis
	**Shapiro–Wilk Normality Test**	
Null Hypothesis	Normally distributed data	
Shapiro–Wilk statistic	0.98387	
*p*-Value	2.20 × 10^−16^	
Conclusion	Reject Null Hypothesis that data are normal	
	**Wilcoxon Signed Rank Test**	
Alt. Hypothesis:	True location is greater than 0	
V	6117396	
*p*-Value	2.2 × 10^−16^	
Conclusion	Reject Null Hypothesis	

**Table 3:** Statistical tests for positive and negative sentiment counts for March 2021. (**a**) Proportion test for positive sentiment. (**b**) Shapiro–Wilk normality test for sentiment scores. (**c**) Wilcoxon signed rank test for mean of sentiment scores.

	Proportion Test	Exact Binomial Test
Null Hypothesis	−ve senti ≥ +ve senti	−ve senti ≥ +ve senti
Alt. Hypothesis	True *p* is less than 0.5	True *p* is less than 0.5
*p*-Value	2.2 × 10^−16^	2.2 × 10^−16^
Conclusion	Reject Null Hypothesis	Reject Null Hypothesis
	**Shapiro–Wilk Normality Test**	
Null Hypothesis	Normally distributed data	
Shapiro–Wilk statistic	0.98343	
*p*-Value	2.20 × 10^−16^	
Conclusion	Reject Null Hypothesis that data are normal	
	**Wilcoxon Signed Rank Test**	
Alt. Hypothesis:	True location is greater than 0	
V	16629414	
*p*-Value	2.20 × 10^−16^	
Conclusion	Reject Null Hypothesis	

**Table 4:** Statistical tests for positive and negative sentiment counts in February and March 2021. (**a**) Tests for change in positive sentiment from February to March. (**b**) Tests for change in negative sentiment from February to March.

	Proportion Test	Exact Binomial Test
Null Hypothesis	Mar’21 +ve senti ≥ Feb’21 +ve senti	Mar’21 +ve senti ≥ Feb’21 +ve senti
Alt. Hypothesis	True *p* is less than 0.5	True *p* is less than 0.5
*p*-Value	0.03262	0.03262
Conclusion	Reject Null Hypothesis	Reject Null Hypothesis
	**Proportion Test**	**Exact Binomial Test**
Null Hypothesis	Feb’21 −ve senti ≥ Mar’21 −ve senti	Feb’21 −ve senti ≥ Mar’21 −ve senti
Alt. Hypothesis	True *p* is less than 0.5	True *p* is less than 0.5
*p*-Value	0.01159	0.01158
Conclusion	Reject Null Hypothesis	Reject Null Hypothesis

**Table 5:** Reasons to decline COVID-19 vaccination in the US (Data source: [13]).

Key Reasons to Affect Vaccine Administration	Percentage
Concerned about possible side effects	20.86
Plan to wait and see if it is safe	17.04
Do not trust COVID-19 vaccines	12.55
Do not trust the government	9.63
Do not believe I need a vaccine	8.32
Do not know if a vaccine will work	8.32
Other people need it more right now	7.11
Do not like vaccines	4.39
Doctor has not recommended it	2.54
Concerned about the cost	1.42
Other	7.15
Did not report	0.68

## Data Availability

Data can be available upon request.

## Abbreviations

The following abbreviations are used in this manuscript:

COVID-19	Coronavirus Disease 2019
CDC	Centers for Disease Control and Prevention
NPIs	Non-pharmaceutical interventions
HPS	Household Pulse Survey
PSS	Public Sentiment Scenarios
